# Selective Capture and Purification of MicroRNAs and Intracellular Proteins through Antisense-vectorized Magnetic Nanobeads

**DOI:** 10.1038/s41598-019-39575-7

**Published:** 2019-02-14

**Authors:** Isabel Gessner, Xiaojie Yu, Christian Jüngst, Annika Klimpel, Lingyu Wang, Thomas Fischer, Ines Neundorf, Astrid C. Schauss, Margarete Odenthal, Sanjay Mathur

**Affiliations:** 1 Institute of Inorganic Chemistry, University of Cologne, Greinstr. 6, 50939 Cologne, Germany; 2 Institute for Pathology, University Hospital of Cologne, Kerpener Str. 62, 50924 Cologne, Germany; 3 Center for Molecular Medicine (CMMC), University of Cologne, Robert-Koch-Straße 21, 50931 Cologne, Germany; 4 Cluster of Excellence - Cellular Stress Responses in Aging-Associated Diseases (CECAD), Imaging Facility, University of Cologne, Joseph-Stelzmann-Str. 26, 50931 Cologne, Germany; 5 Institute of Biochemistry, University of Cologne, Zuelpicher Str. 47, 50674 Cologne, Germany; 6 Center of Integrative Oncology, University Clinic of Cologne and Bonn, Cologne and Bonn, Germany

## Abstract

MicroRNAs (miRNAs) are small non-coding nucleotides playing a crucial role in posttranscriptional expression and regulation of target genes in nearly all kinds of cells. In this study, we demonstrate a reliable and efficient capture and purification of miRNAs and intracellular proteins using magnetic nanoparticles functionalized with antisense oligonucleotides. For this purpose, a tumor suppressor miRNA (miR-198), deregulated in several human cancer types, was chosen as the model oligonucleotide. Magnetite nanoparticles carrying the complementary sequence of miR-198 (miR-198 antisense) on their surface were delivered into cells and subsequently used for the extracellular transport of miRNA and proteins. The successful capture of miR-198 was demonstrated by isolating RNA from magnetic nanoparticles followed by real-time PCR quantification. Our experimental data showed that antisense-coated particles captured 5-fold higher amounts of miR-198 when compared to the control nanoparticles. Moreover, several proteins that could play a significant role in miR-198 biogenesis were found attached to miR-198 conjugated nanoparticles and analyzed by mass spectrometry. Our findings demonstrate that a purpose-driven vectorization of magnetic nanobeads with target-specific recognition ligands is highly efficient in selectively transporting miRNA and disease-relevant proteins out of cells and could become a reliable and useful tool for future diagnostic, therapeutic and analytical applications.

## Introduction

Bioconjugated nanoparticles are suitable probes for oligonucleotide detection, transport and their controlled release, useful for both biomolecular detection as well as therapeutic applications. While most of these structures have so far focused on the delivery and sensing of DNA molecules, RNA nanotechnology has gained momentum due to the diverse and versatile nature of oligonucleotide-nanoparticle conjugates ranging from self-assembled RNA nanoparticles^1^, to organic and inorganic platforms which are used as transporters for RNA molecules^2^. MicroRNAs (miRNAs), small endogenous non-coding RNAs, play an important role in posttranscriptional regulation and are thus promising candidates for tailored therapeutic targeting. A vast variety of human genes is known to be regulated by miRNAs based on their complementary sequence, which leads to the suppression of protein translation^3^. Recent advances in the identification of gene-specific miRNAs has opened a new field of cancer therapy based on their targeted transport *via* nanocarriers, however the mechanisms underlying nanoconjugate-induced gene expression are not fully understood^4^. On the other hand, iron oxide nanoparticles (IONPs) have been extensively studied to probe nano-bio interactions majorly due to their low cytotoxicity and facile strategies known for their surface functionalization. Bioconjugated IONPs have been used as delivery vehicles for miRNAs and have been tested for hyperthermic treatments of cancer cells^5^ or the visualization of the transporters *via* magnetic resonance imaging (MRI)^6,7^. Moreover, the magnetic nature of iron oxides offers magnetic separation of biomolecules including cells^8^, proteins^9^ and nucleotides^10^ that simplifies post-detection assays. However, the specific recognition of miRNAs in physiological environment is still a major challenge considering their small size and structural similarity.

Thus, we have used IONPs with surface immobilized antisense miRNA as effective probes for intracellular capturing and purification of miRNA and associated proteins. The effectiveness of our approach was demonstrated using miR-198 as the probe molecules that enabled extraction of proteins out of hepatocarcinoma cells. Moreover, the identified proteins indicated a stress-responsive release pathway of the tumor-suppressor oligonucleotide, which in conjunction with their selective capture can significantly enhance our capabilities in early diagnosis of cancer or in monitoring the therapeutic efficacy of the given treatment.

## Results

### Characterization of custom-made magnetic beads

**Figure 1:**
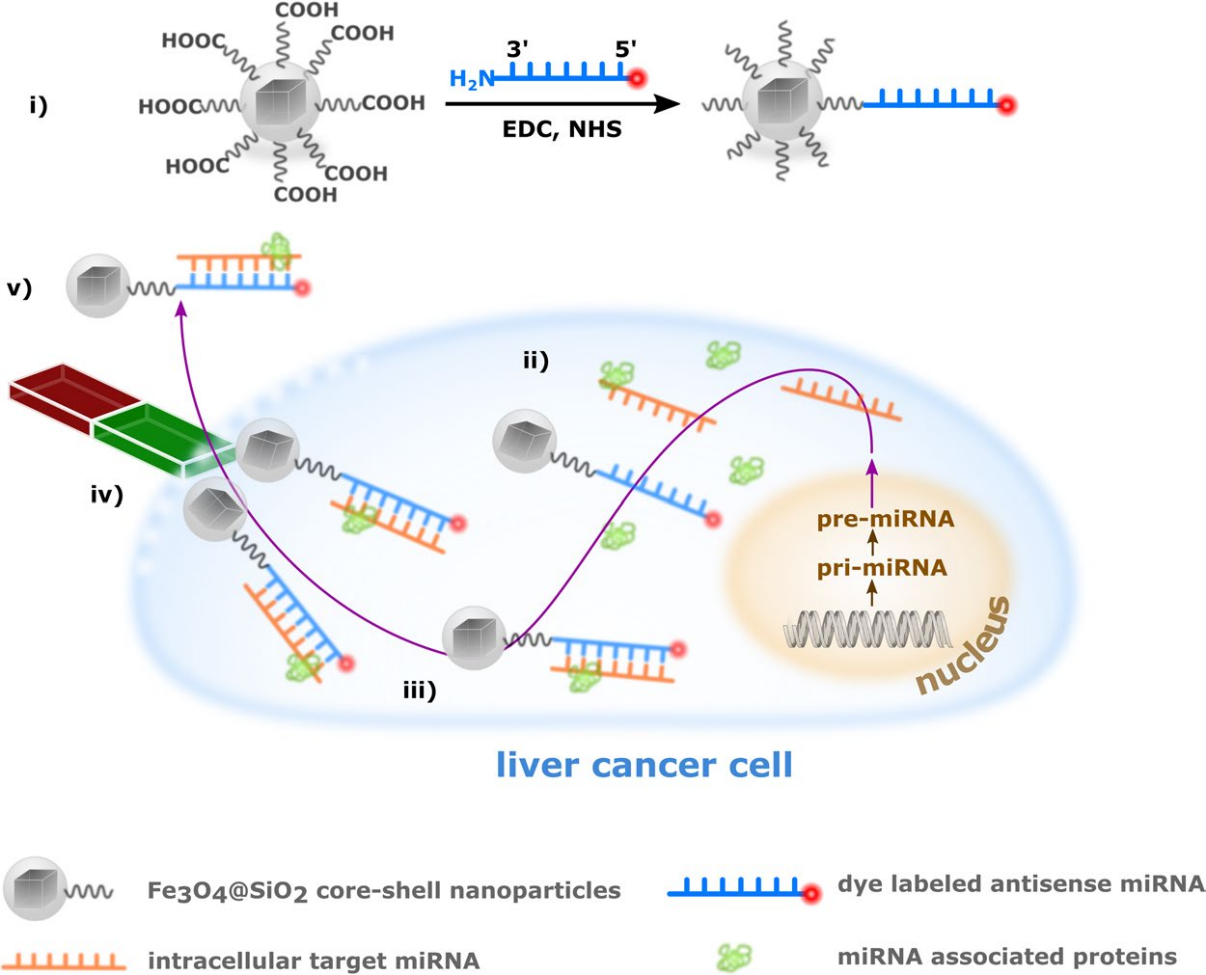
Schematic outline of the synthesis of miR-198 antisense functionalized magnetic beads and their use for the selective capturing of miR-198 and associated proteins out of liver cancer cells: (i) Nanoparticle synthesis and surface modification is followed by (ii) their cellular uptake and (iii) the selective capturing of miR-198. (iv) Upon cell lysis and magnetic separation, (v) quantification of miR-198 capturing efficiency and identification of attached proteins via mass spectrometry can be performed.

Transformation of isotropic nanoparticles into cell-interrogating vectors requires appropriate surface affinity created by attachment of specific biomolecular probes such as cell-penetrating peptides, oligonucleotides or aptamers. For the cellular extraction and purification of miRNAs and proteins, silica-coated magnetite nanoparticles (Fe_3_O_4_@SiO_2_) were functionalized with citric acid to obtain nanobeads with intractable carboxylic surface termination that was used for the covalent attachment of an antisense miRNA (miR-198 antisense) following the carbodiimide coupling chemistry (Fig. 1).

The successful internalization of surface-functionalized beads by liver cancer cells was followed by the intracellular selective capturing of miR-198 and associated proteins, which could be magnetically separated from the cells in facile manner after lysis of the cellular membrane. Dye labeled oligonucleotides used in this work allowed their intracellular tracking *via* confocal microscopy.

**Figure 2:**
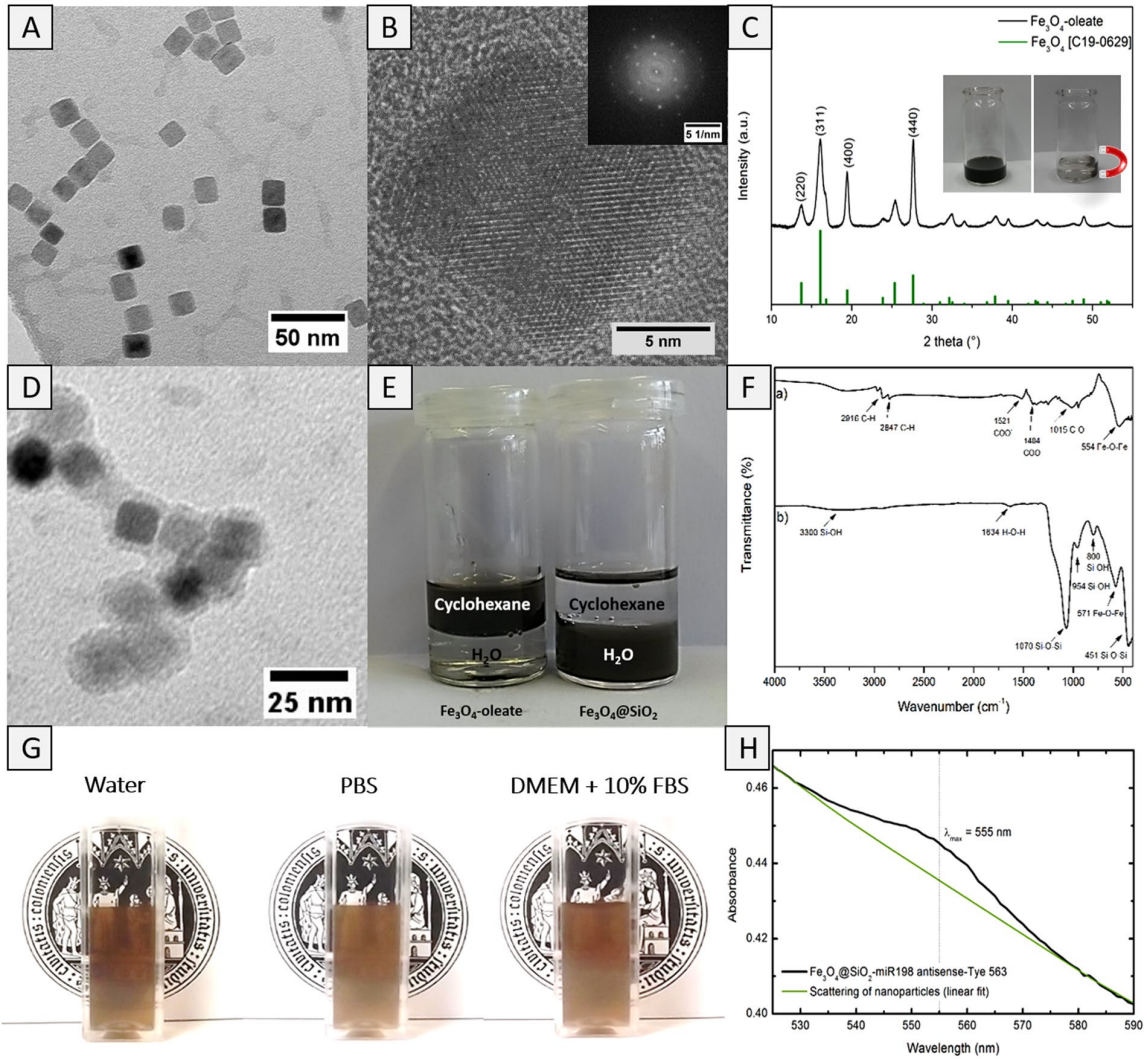
(**A**) TEM image of cube-shaped magnetite nanoparticles prepared through a thermal decomposition with oleic acid as surface active ligand. (**B**) High resolution TEM of cube-shaped magnetite nanoparticles, inset shows the electron diffraction pattern of a single crystal. (**C**) XRD of oleate-capped magnetic beads compared to cubic magnetite (JCPDS file no C19-0629). The small inset demonstrates that as-prepared particles can be easily separated out of a solvent via a magnet. (**D**) TEM image of SiO_2_ coated magnetite NPs. (**E**) Oleate-capped magnetite NPs turn from hydrophobic to hydrophilic after the surface modification with SiO_2_. (**F**) IR spectra of (a) oleate-capped magnetic beads and (b) silica coated magnetic beads. (**G**) Photographs of dispersions of Fe_3_O_4_@SiO_2_ core-shell nanoparticles in distilled water, PBS and cell culture medium containing 10% serum proteins. (**H**) UV-vis measurement of Fe_3_O_4_@SiO_2_-miR-198 antisense-Tye563 construct (black). A linear fit was performed to underline the scattering of the particles (green).

Homogeneous magnetite nanoparticles with a mean size of 14.6 ± 1.1 nm (TEM images, Fig. 2A) were obtained by controlled thermal decomposition of the iron-oleate precursor (Fe(C_18_H_33_O_2_)_3_) in high boiling solvents. As-obtained particles displayed cube-shaped morphology and high crystallinity as verified by high-resolution transmission electron microscopy (Fig. 2B) and X-ray diffraction measurements (Fig. 2C). The X-ray diffraction peaks exhibited the formation of cubic Fe_3_O_4_ phase (JCPDS file no. C19-0629) that showed high magnetization useful in their separation from biological milieu using a magnet (Fig. 2C, inset).

Due to the hydrophobic nature of oleic acid-functionalized nanoparticles, a surface modification with silica overlayer was performed to reverse the wetting behavior that rendered the magnetic beads hydrophilic. For this purpose, a modified micro-emulsion method was employed using IGEPAL-C520 as surfactant and Si(OEt)_4_ (tetra-ethyl ortho-silicate, TEOS) as precursor for the controlled deposition of SiO_2_ coating onto the magnetite nanoparticle surface. The successful formation of a conformal silica shell was evident in the TEM analyses that revealed a homogeneous nanometric coating (thickness 3 nm, Fig. 2D). Owing to their hydrophilic exterior, the Fe_3_O_4_@SiO_2_ core-shell particles could be stabilized in aqueous environment over a period of several days, which is a crucial requirement for cellular studies (Fig. 2E,G). The FT-IR measurements displaying typical Fe-O-Fe stretches (554 and 571 cm^−1^)^9^ and C-O (1015 cm^−1^)^11^ and C-H vibrations (2847 cm^−1^ and 2916 cm^−1^) due to oleate groups confirmed both chemical composition and topology of pristine and functionalized nanoparticles. The typical asymmetric and symmetric stretches due to carboxylate groups (1521 and 1404 cm^−1^) confirmed the ligation of carboxylate units. After the silica coating, Si-O-Si stretches at 1070 cm^−1^ and 451 cm^−1^ as well as Si-OH oscillation bands at 954 cm^−1^ and 800 cm^−1^ were observed (Fig. 2F)^12^. Moreover, the broad band around 3300 cm^−1^ was assigned to surface silanol groups that are responsible for the high hydrophilicity of the particles. The successful modification of hydroxyl functionality in Fe_3_O_4_@SiO_2_ core-shell particles was verified by measuring the zeta-potential that changed to more negative values from −24.6 ± 1.1 mV to −45.7 ± 0.9 mV due to deprotonation of carboxylic acid groups attached to the particle surface. The hydrodynamic diameter of as-prepared nanoparticles measured by dynamic light scattering was significantly larger (86.2 ± 10.2 nm) than the particle sizes observed in TEM images (ca. 15 nm, Fig. 2A) probably due to the presence of hydrophilic carboxylic acid groups that increased the thickness of hydration shell due to hydrogen bonding. Finally, the miR-198 antisense-functionalized NPs were prepared by employing the carbodiimide coupling chemistry that was indicated by a post-synthesis change in zeta potential, which increased to −23.2 ± 0.4 mV due to the replacement of carboxylic acid groups by the oligonucleotide. UV-vis measurements performed upon magnetically separated particles proved the successful attachment of the oligonucleotide. Besides the typical scattering of nanoparticles, an absorbance with maximum intensity around 555 nm could be observed due to the characteristic absorbance of Tye563 dye (Fig. 2H).

### Cellular uptake and quantification of captured RNA

**Figure 3:**
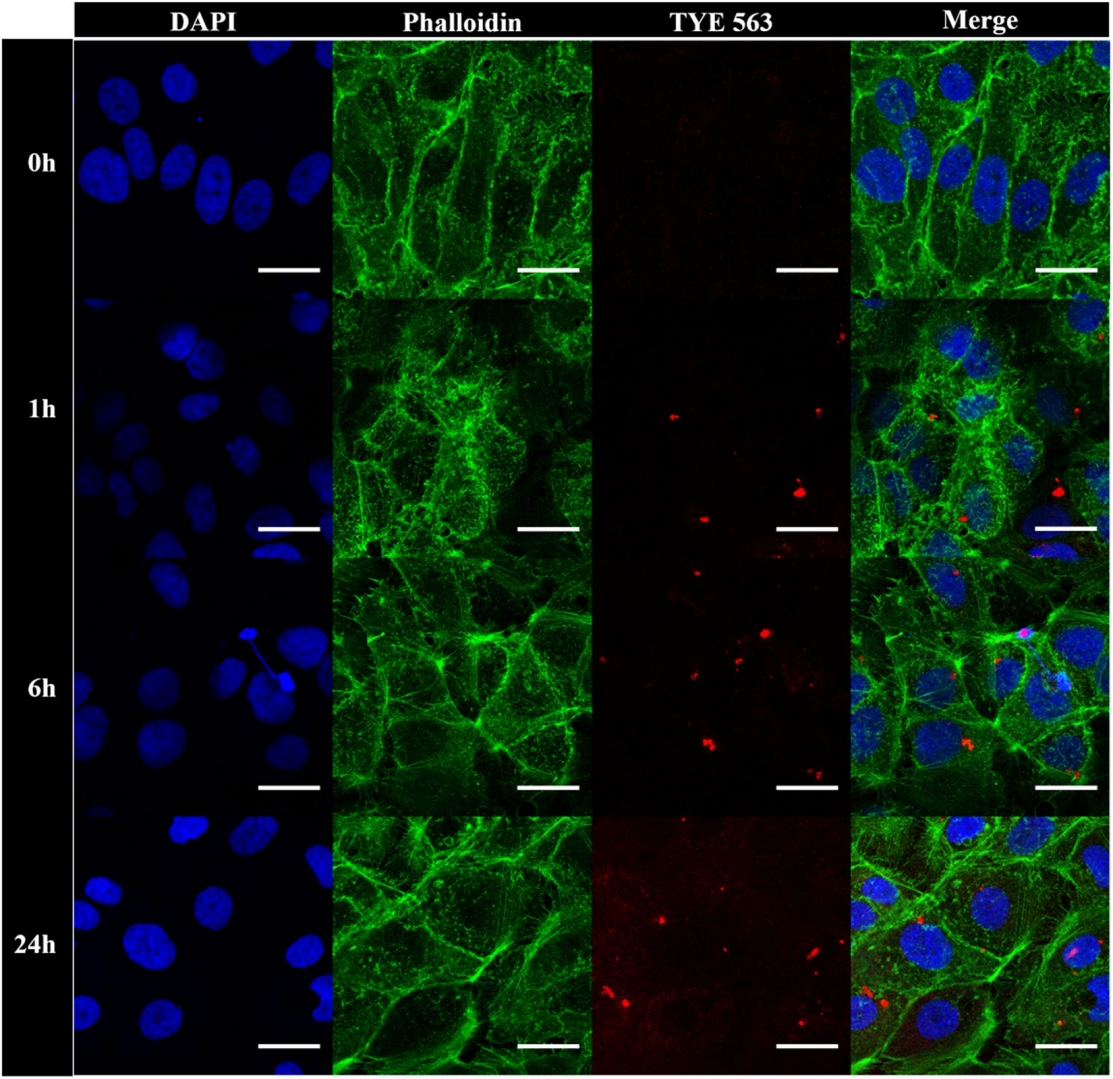
Cellular uptake of miR-198 antisense functionalized magnetic beads into Huh7 cells. Images were taken before and after 1, 6 and 24 hours incubation time followed by cell staining and fixation. The scale bar in all images refers to 25 µm.

**Figure 4:**
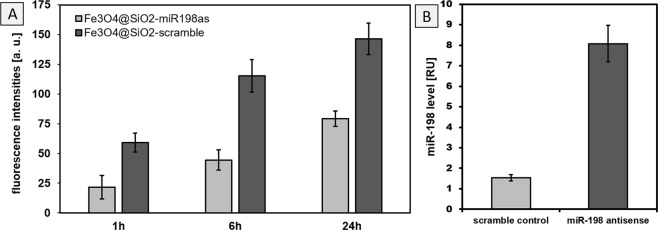
(**A**) Quantitative cellular uptake of functionalized magnetic beads into Huh7 cells after 1, 6 and 24 h incubation time, respectively. (**B**) Relative levels of miR-198 captured by scramble coated versus miR-198 antisense coated nanoparticles. miR-198 levels were determined by real time PCR, after their capturing through miR-198 antisense and scramble functionalized magnetic beads.

The nanoparticles coated with miR-198 antisense were investigated towards their cellular uptake and protein capture capacity. Uptake was performed using Huh7 hepatoma cells, transgenically expressing miR-198 driven by the tet-on promoter system^13^. After 24 hours incubation time, cells were stained with DAPI and phalloidin to label the cell nucleus and the cytoskeleton of the cells, respectively (Fig. 3). Although the red emission of dye-marked beads was already visible after an incubation time of one hour, confocal images displayed an enhanced intensity with increasing incubation time (6 and 24 hours) indicating the successful accumulation of magnetic beads over time. Additional orthogonal views of confocal microscopic images demonstrated that the emission of dye labeled particles occurred mainly in the Z-disk of the phalloidin counterstained cytoskeleton and thus indicated the internalization of conjugates (Fig. S1). This was further supported by flow cytometric studies, that validated a time-dependent increase in uptake of the miR198 antisense conjugate, as well as of a control sample of nanoparticles functionalized with a scrambled oligonucleotide (Figs 4A and S2). In general, no adverse effects were visible on the cells after incubation (24 hours) with magnetic beads, which suggested the low cytotoxicity of bioconjugated nanoparticles.

After cell lysis, magnetic beads with surface-attached biomolecules were isolated through magnetic separation and real time PCR was performed to determine the miR-198 capturing ability of both conjugates. Comparative analyses and quantitative evaluations confirmed five-fold higher capturing efficiencies for miR-198 antisense modified nanoparticles (Fig. 4B) demonstrating the high efficacy of the proposed analytical model.

### Identification of captured proteins

In this work, a model miRNA, miR-198, was chosen since it has been identified as tumor suppressor in hepatocellular carcinoma inhibiting cell growth and proliferation^14^. Interestingly, during liver diseases, increased miR-198 levels can be found in the blood stream while the intracellular expression is significantly downregulated. It has been recently revealed that miR-198 is actively transported out of liver cancer cells *via* exocytosis^15^, although proteins responsible for this transport are yet unknown. Therefore, we also identified proteins attached to miR-198 capturing beads that provided new insights to elucidate the bioanalytical potential of this new process. All proteins attached to miR-198 antisense modified particles were eluted and subjected to mass spectrometry for identification purposes. Table 1 provides an overview of 33 proteins that could be identified and have only been found in the presence of miR-198 carriers, not on control particles, so that the non-specific adsorption of proteins such as those that may form a protein corona could be excluded.

**Table 1:** List of proteins which could be eluted from miR-198 antisense conjugated beads, identified by mass spectrometry.

Gene	Encoding protein	Gene	Encoding protein
RPL37A	Ribosomal protein L37a	BAG2	BCL2 associated athanogene 2
NDUFA4	Mitochondrial complex associated	ZNF444	Zinc finger protein 444
TOMM20	Translocase of outer mitochondrial membrane 20	SRRT	Serrate, RNA effector molecule homolog
AKR1D1	Aldo-keto reductase family 1 member D1	EIF3A	Eukaryotic translation initiation factor 3 subunit A
HABP4	Hyaluronan binding protein 4	SPATS2L	SPATS2-like protein
ZNF207	Zinc finger protein 207	DNAJB1	DnaJ homolog subfamily B member 1
TARDBP	TAR DNA binding protein	MCM5	DNA helicase;DNA replication licensing factor MCM5
PEBP1	Phosphatidylethanolamine binding protein 1	PCOLCE2	Procollagen C-endopeptidase enhancer 2
SORD	Sorbitol dehydrogenase	CALR	Calreticulin
VAPA	Vesicle-associated membrane protein, associated protein A	EHD3	EH domain-containing protein 3
MAPRE2	Microtubule associated protein RP/EB family member 2	MCM3	DNA replication licensing factor MCM3
SPATS2L	Spermatogenesis associated serine rich 2 like	NSUN5	Probable 28S rRNA (cytosine-C(5))-methyltransferase
DNAJB1	DnaJ heat shock protein family (Hsp40) member B1	EIF4G2	Eukaryotic translation initiation factor 4 gamma 2
MCM5	Minichromosome maintenance complex Pomponent 5	CKAP5	Cytoskeleton-associated protein 5
PCOLCE2	procollagen C-endopeptidase enhancer 2	TJP2	Tight junction protein ZO-2
RBM12B	Germinal-center associated nuclear protein	HSDL2	Hydroxysteroid dehydrogenase-like protein 2
RPSA	Ribosomal protein SA		

**Figure 5:**
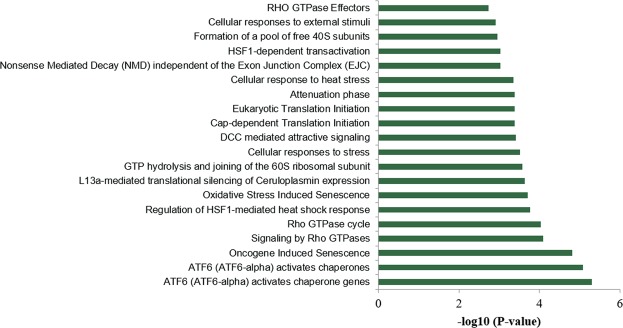
Cellular pathways in which eluted proteins from miR-198 antisense conjugated beads are involved. The higher the P-value, the higher the number of proteins involved in this pathway.

The high number of identified proteins included ribosomal proteins as well as zinc finger proteins and several enzymes. Detected proteins are responsible for several different cell signaling pathways, although the majority of proteins could be assigned to stress induced responses, like activating transcription factor 6 (ATF6) signaling (Fig. 5). ATF6 is known as a transmembrane protein that is activated through endoplasmic reticulum (ER) stress^16^.

## Discussion

**Figure 6:**
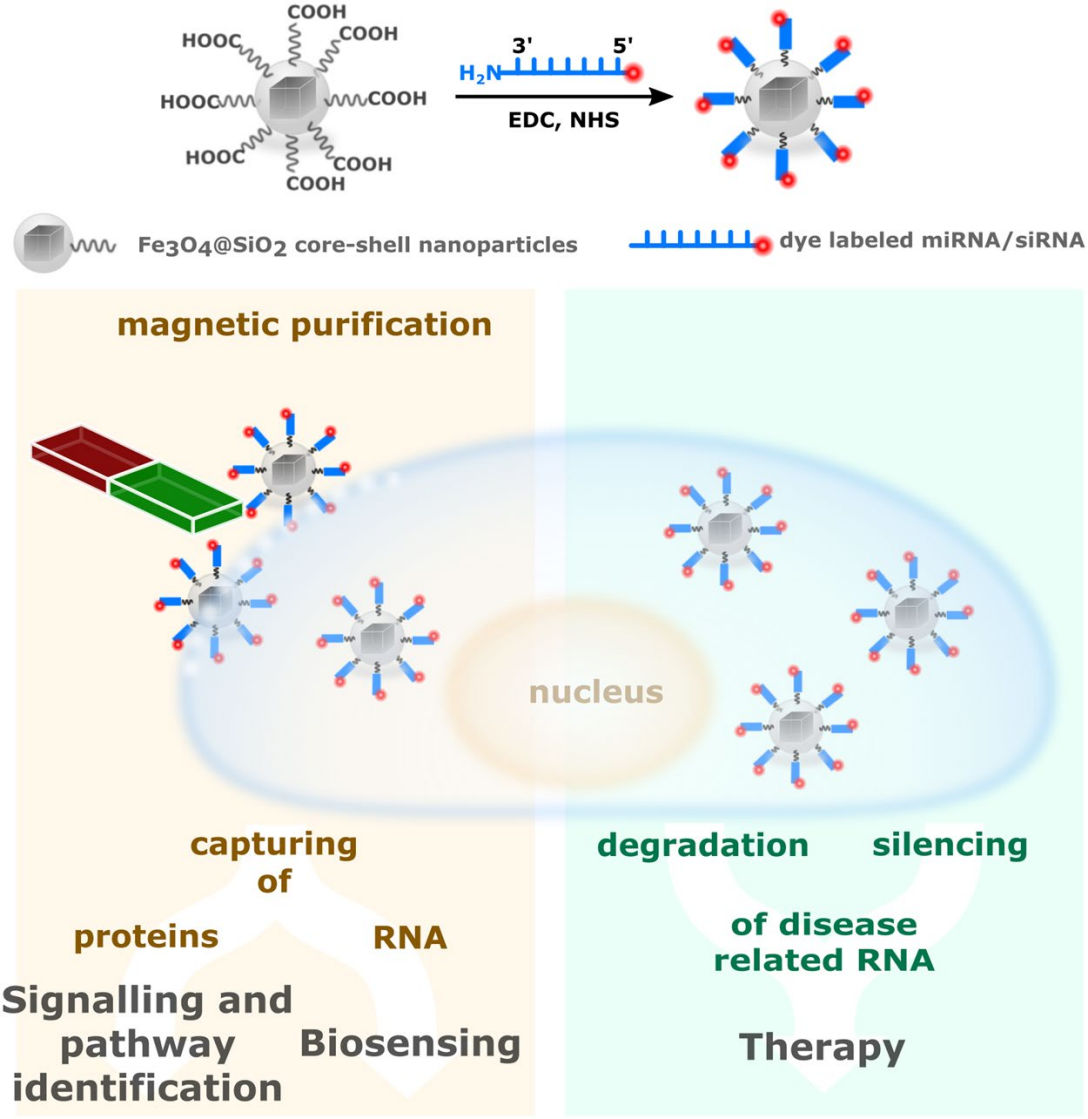
The versatile potential applications of RNA functionalized magnetic beads, as prepared in this work.

The employment of RNA antisense functionalized nanoparticles as versatile intracellular delivery systems shows promising and useful therapeutic applications. Current research regarding RNA functionalized nanoparticles is highly focused on therapeutic applications considering RNA interference, for instance by silencing of a disease promoting miRNA through controlled strain alignment and inhibition of protein translation^7^. This is usually based on the surface immobilization and transport of short interfering RNA (siRNA) that typically results in the degradation of desired RNA strains^17^. Although free siRNAs have been administered as therapeutic agent to cells, their cellular uptake efficiency is very low but has been shown to be significantly enhanced when conjugated to nanoparticle-based carriers^18^. Recently, first deliveries of siRNA-nanoparticle conjugates in humans have been proven very successful for the treatment of cancers acting as a promising indicator for developing new concepts for early detection and new therapeutic protocols against cancer^19^. Indeed, the strategy described in this work for the surface functionalization of magnetic nanoparticles is not specific for antisense miRNA and could be analogously extended for the attachment of other oligonucleotides such as siRNA. However, the therapeutic application of miRNA-conjugated magnetic beads was beyond the scope of this work. Instead, we demonstrate that surface-vectorization with appropriate biomolecules is suitable for the selective separation of miRNA out of cells and their magnetic purification, using miR-198 as a model nucleotide. In view of the above, this method holds great potential not only for the intracellular extraction of oligonucleotides but also for biosensing applications. Moreover, our data demonstrated that this approach could additionally be used for the capturing and identification of specific proteins and their classification into cell signaling pathways (Fig. 6). Considering the small size and sequence similarity of miRNAs, the detection and selective separation of a specific miRNA out of cellular environment consisting of a vast number of other biomolecules is a challenging avenue and the high sensitivity of custom-made magnetic beads used in this work points out the unexplored potential of nanoparticles in configuring posttranscriptional mechanisms. Although few reports exist on the detection of miRNAs using magnetic particles, most of them involve micro-structured particles and their application in biosensing, e.g., in the immobilized form on a sensor stripe. The magnetic capturing abilities are generally tested out of a standardized miRNA solution with known concentration or in prepared RNA extracts from cells. Only few cases are known were separation was performed in the presence of other biomolecules, however to the best of our knowledge, capture studies of miRNA in cancer cells using functionalized magnetic beads have not yet been reported.

Customized magnetic beads used in this work were not only highly stable in biological environment, but also possessed high miR-198 capturing efficiencies after being modified with miR-198 antisense strains. Since miR-198 is a tumor suppressor in hepatocellular carcinoma and has been shown to be actively transported out of cells during liver cancer development, our work additionally focused on the identification of responsible proteins. Mass spectrometric studies on detected proteins revealed that their intracellular function is closely related to stress-related, e.g., ER-stress associated pathways. As the ER is a quality control system which is responsible to ensure a correct protein folding, unfolded or misfolded proteins are subjected to degradation. In case of ER stress, unfolded proteins accumulate in the ER leading to the activation of the unfolded protein response (UPR), which is triggered through proteins such as ATF6, to restore ER homeostasis^20^. Similar to UPR, autophagy is a cellular self-recycling process that disassembles unnecessary or dysfunctional proteins or components^21^. Although, the interplay between ATF6 and autophagy is only poorly understood to date, accumulated evidences emphasize that both pathways are related to each other regarding to the counterbalance of ER stress^22^. This indicates that stress-mediated pathways such as autophagy might play a crucial role in the secretion of miR-198, however, further studies on this topic are needed to support these results. In summary, this work demonstrates the enormous potential of RNA functionalized magnetic nanoparticles as vehicles for two-way cellular transport that could be potentially exploited to specifically deliver therapeutic RNAs to cancer sites thus making them indispensable for future medicinal approaches.

## Methods

### Chemicals

All chemicals were used without further purification. Iron(III)-chloride hexahydrate (99%) was procured from Merck, sodium oleate, IGEPAL C520, tetraethyl orthosilicate (98%), 1-ethyl-3-(3-dimethylaminopropyl) carbodiimide (EDC) hydrochloride, N-hydroxysuccinimide (NHS), ammonium hydroxide (28–30%) as well as the Trizol reagent were obtained from Sigma Aldrich and 1-octadecene (90%) and oleic acid (90%) were received from Alfa Aesar. Roti®-Mount FluorCare DAPI solution was procured from Roth. The reverse transcription and polyadenylation reagents as well as miR-198 specific primers sets were purchased from Qiagen. Mastermix reagents for real time PCR were obtained from Promega. Urea, dithiothreitol (DTT), iodoacetamide (IAA), doxycycline, lipofectamine, G418, and hydromycine were purchased from Thermo Fisher.

### Oligonucleotides

Custom-made 5′Tye563-labeled and 3′amino modified oligonucleotides were ordered through Exiqon. Two different types of oligonucleotides were used in this work: miR-198 antisense (3′ AmMC6–GAACCTATCTCCCCTCTGGACC-Tye563) and a control (scramble) sequence (3′ AmMC6-GTGTAACACGTCTATACGCCCA-Tye563). AmMC6 represents an NH_2_ group with C_6_ linker while Tye563 is a dye which has its absorption maximum at 549 nm. In order to avoid the quick degradation by nucleases, Locked Nucleic Acids (LNAs) were used.

### Synthesis of magnetic nanoparticles

Oleate capped magnetite nanoparticles were prepared by slightly modifying a previously described method^23^. In a typical procedure, an iron oleate precursor was prepared by dissolving 40 mmol (10.8 g) FeCl_3_ · 6 H_2_O and 120 mmol (36.5 g) sodium oleate in a solvent mixture of 140 ml cyclohexane, 80 ml ethanol and 60 ml distilled water. The solution was stirred for four hours at 70 °C. After cooling down to room temperature, the organic phase containing the precursor was separated using a separatory funnel and washed three times with distilled water. The product was finally obtained by removal of the solvent under vacuum. For the thermal decomposition of the iron oleate precursor, 5.0 mmol (4.5 g) of iron oleate were dissolved in 98.2 mmol (25 g) of 1-octadecene followed by the addition of 5.0 mmol (1.43 g) of oleic acid. The resulting mixture was then degassed under vacuum at 100 °C for 30 minutes before heating it up to 315 °C for another 30 minutes. After cooling down to room temperature, the obtained black particles were precipitated using 60 ml ethanol and collected *via* centrifugation at 11000 rpm for one hour. The particles were extensively washed with a mixture of cyclohexane/ethanol (1:3 vol %) and finally dried under vacuum.

### Synthesis of Fe_3_O_4_@SiO_2_ nanoparticles

In order to render the oleate-capped particles hydrophilic, a reverse micro-emulsion method was used as described in the literature^24^. For this purpose, 25 mg of previously prepared oleate-capped iron oxide nanoparticles were dispersed in 160 ml cyclohexane under sonication before a mixture of 8 ml IGEPAL solution and 25 ml cyclohexane were added under stirring. Then, 125 µl of ammonium hydroxide were added to the dispersion followed by sonication for 30 minutes. Following this, 1 ml tetraethyl orthosilicate (TEOS) were added in small aliquots of 100 µl over 20 hours to obtain a homogenous silica coating. Fe_3_O_4_@SiO_2_ nanoparticles were obtained *via* centrifugation (11000 rpm, 20 minutes) and washed (3 times) with ethanol and finally dried under vacuum.

### Synthesis of Fe_3_O_4_@SiO_2_-COOH nanoparticles

Carboxylic acid groups were linked to the surface of silica coated magnetite nanoparticles by dispersing 25 mg of Fe_3_O_4_@SiO_2_-NPs in 6 ml of an ethanol water mixture (1:1) followed by sonication for 30 minutes. The dispersion was heated to 85 °C under reflux and subsequently 11 mg of citric acid monohydrate which were dissolved in 4 ml of the same solvent mixture were added. The reaction was maintained at 85 °C for one hour and then cooled to room temperature. The as-obtained particles were collected *via* magnetic separation and washed with PBS buffer. For characterization purposes, the particles were dried under vacuum to obtain a black powder.

### Covalent attachment of H_2_N-miR-198 antisense on Fe_3_O_4_@SiO_2_-COOH nanoparticles

The miR-198 antisense strain was covalently attached to the surface of magnetic nanobeads *via* carbodiimide coupling. In a typical procedure, 100 µl of a 31 mM solution of EDC hydrochloride and 100 µl of a 0.35 M solution of NHS were prepared in distilled water. Moreover, a stock solution of 1.8 mg of Fe_3_O_4_@SiO_2_-COOH-NPs in 1 ml sterile water was prepared. 200 µl of the particle dispersion were then mixed with the EDC solution to activate the terminal carboxylic acid groups. In order to enhance the efficiency of this reaction, NHS was added as well as 300 µl of distilled water to increase the reaction volume and ensure a good mixing. After stirring for one hour, 100 µl of a 10 µM miR-198 antisense solution was added to obtain a ratio of 100:1- for miR-198 antisense/nanoparticle and the mixture was stirred overnight in the dark. miRNA functionalized nanoparticles were collected *via* magnetic separation and the product was obtained after several washing steps with sterile water. The same procedure was used for the coupling of the control oligonucleotide (scramble).

### Particle characterization

Size and morphology of the particles were analyzed *via* transmission electron microscopy (TEM) studies using a ZEISS Leo912 microscope operated at an acceleration voltage of 120 kV. For sample preparation, diluted dispersions of the samples were prepared in ethanol, dropped onto a carbon-covered standard TEM grid (QUANTIFOIL Multi A) and dried under air. Phase purity and crystallinity of the samples was measured using a powder STOE-STADI MP X-Ray diffractometer (XRD) with Mo radiation (λ = 0.7093 Å) and measured peak patterns were compared to reference JCPDS files. Infrared spectra were acquired using a Perkin Elmer Fourier transform infrared (FTIR) spectrometer between 400 and 4000 cm^−1^. UV-vis spectroscopy (LAMBDA 950 Perkin Elmer) was employed to detect the dye-labeled miRNA on the particle surface. Zeta potential and hydrodynamic diameter of the particles were measured using aqueous dispersions (distilled water with a pH of 6.5) of the particles employing a Malvern Zetasizer NanoZS.

### Cell Studies

Huh7 cells (kindly provided by Peter Schirmacher and Kai Breuhahn, University of Heidelberg, Germany) were cultured in 10% FBS DMEM medium at 5% CO_2_ and 37 °C. Cells were stably transfected with pTet-on advanced plasmid and pmRi-ZsGreen1-miR-198 plasmid using lipofectamin. After G418 and hygromycin selection, Huh7 stable cells (Huh7-ZsGreen1-miR-198), harboring doxycycline inducible miR-198 expression system, were established.

Huh7-ZsGreen1-miR-198 cells were seeded at a density of 3000 cells/well in 8-well µ-slide chambers and grown overnight at 37 °C and 5% CO_2_. Then, medium was removed and cells were washed with PBS twice before the addition of nanoparticles. 5.4 µg miR-198 antisense functionalized beads were dispersed in 400 µl cell culture medium and added to the cells and doxycycline was added at a final concentration of 1 ng/ml. After incubation for 24 hours, cells were washed twice with PBS followed by 4% PFA fixation. After 15 minutes, cells were permeabilized with 0.1% Triton-X100. For cell membrane staining, phalloidin was diluted in PBS to 1:40, 100 µl were added to each well and cells were incubated for 15 minutes in the dark. Cells were washed again with PBS and mounted using Roti®-Mount FluorCare DAPI solution. Confocal microscope images were acquired using a confocal laser scanning microscope (TCS, SP8, Leica Microsystems) with 40x air objective and a numerical aperture of 0.85.

### Quantitative cellular uptake

To investigate the cellular uptake quantitatively, flow cytometry experiments were performed. Therefore, Huh7 cells were seeded in 24-well plates and grown to sub-confluency. Before treatment of the cells, medium was removed and cells were washed twice with PBS. Afterwards, 8.1 µg functionalized beads were dispersed in 400 µl serum-containing medium and added for 1, 6 or 24 hours to the cells. After the incubation, cells were washed twice with PBS, detached with phenol red-free trypsin and re-suspendend in phenol red-free medium. Cellular uptake was determined with the guava easyCyteTM System (Merck) using the RED-B (695/50) channel, counting 10,000 cells per well. All measurements were performed twice in triplicates.

### Captured RNA and protein purification

After nanoparticle uptake, cells were washed twice with cold PBS, and lyzed in lysis buffer (150 mM NaCl, 25 mM TrisHCl, 5 mM EDTA, 0.5% NP40, 0.1 mM PMSF, pH 7.6) and further sonicated for particle release. Magnetic beads were separated from cell debris by magnetic force and were subsequently washed three times with cold lysis buffer and cold PBS using a magnetic rack. Proteins or RNA were then isolated. RNA isolation was carried out by using TRIZOL reagent according to the supplier’s instructions. For later normalization, before extraction 2 pmol SV-40 spike-in small RNA was added per 100 µl volume. Proteins were prepared for mass spectrometry by the “In Solution Digest” method. Firstly, the proteins were reduced using 5 mM dithiothreitol (DTT) followed by alkylation using 10 mM iodoacetamide (IAA). Subsequently, proteins were denatured by dissolving them in 2 M urea dissolved in a 50 mM triethylammoniumbicarbonate buffer, and then digested by the trypsin and Lys-C endoproteinase treatment, both used in 1:100 protein/enzyme ratio.

### Mass spectrometry analysis of proteins

Protein samples were applied to the 2D-LC-MS mass spectrometer (Shimadzu) using the LTQ Orbitrap Discovery MS (Thermo Fisher Scientific; Waltham, MA, USA) in collaboration with the mass spectrometric service unit of the CECAD at the University of Cologne. Proteins identified by p-value < 0.05 and fold change (fc) higher than 2-fold in comparison to the scramble control were selected and further analyzed by the reactome pathway analysis (https://reactome.org/).

### miR-198 quantification by real time PCR

RT-PCR miRNA quantification was performed with RNA population extracted from cells as well as on nanoparticles. RNA was polyadenylated and reverse transcribed by means of the miScript II transcriptase. Real time PCR was then performed in triplicates using the miR-198 and the SV-40 primer set following the manufacturer’s instructions. PCR assays were proven for linearity of quantification using a standard curve covering at least two log-fold dilutions. The spiked-in SV-40 RNA was used for normalization and miR-198 values were calculated according to the ΔΔCt method.

## Supplementary information

Supplementary Information

## References

[1] Guo P (2010). The Emerging Field of RNA Nanotechnology. Nat. Nanotechnol..

[2] Xu H, Li Z, Si J (2014). Nanocarriers in Gene Therapy: A Review. J. Biomed. Nanotechnol..

[3] Shenouda SK, Alahari SK (2017). MicroRNA function in cancer: Oncogene or a tumor suppressor?. Sci. Rep..

[4] Fernandez-Piñeiro I, Badiola I, Sanchez A (2017). Nanocarriers for microRNA delivery in cancer medicine. Biotechnol. Adv..

[5] Yin PT, Shah BP, Lee K-B (2014). Combined Magnetic Nanoparticle-based MicroRNA and Hyperthermia Therapy to Enhance Apoptosis in Brain Cancer Cells. Small.

[6] Li Y (2017). Co-delivery of microRNA-21 antisense oligonucleotides and gemcitabine using nanomedicine for pancreatic cancer therapy. Cancer Sci..

[7] Leder A (2015). Micron-sized iron oxide-containing particles for microRNA-targeted manipulation and MRI-based tracking of transplanted cells. Biomaterials.

[8] Xu H (2011). Antibody conjugated magnetic iron oxide nanoparticles for cancer cell separation in fresh whole blood. Biomaterials.

[9] Ilyas S, Ilyas M, Van Der Hoorn RAL, Mathur S (2013). Selective conjugation of proteins by mining active proteomes through click-functionalized magnetic nanoparticles. ACS Nano.

[10] Berensmeier S (2006). Magnetic particles for the separation and purification of nucleic acids. Appl. Microbiol. Biotechnol..

[11] Zhang L, He R, Gu HC (2006). Oleic acid coating on the monodisperse magnetite nanoparticles. Appl. Surf. Sci..

[12] Rubio F, Rubio J, Oteo JL (1998). A FT-IR Study of the Hydrolysis of Tetraethylorthosilicate (TEOS). Spectrosc. Lett..

[13] Gossen M, Bujard H (1992). Tight control of gene expression in mammalian cells by tetracycline-responsive promoters. Proc. Natl. Acad. Sci. USA.

[14] Elfimova N (2013). Control of mitogenic and motogenic pathways by miR-198, diminishing hepatoma cell growth and migration. Biochim. Biophys. Acta - Mol. Cell Res..

[15] Yu X, Eischeid H, Büttner R, Odenthal M (2016). Tumor suppressor microRNA-198 is actively transported out of liver cancer cells. Z. Gastroenterol..

[16] Adachi Y (2008). ATF6 Is a Transcription Factor Specializing in the Regulation of Quality Control Proteins in the Endoplasmic Reticulum. Cell Struct. Funct..

[17] Karp JM, Peer D (2018). Focus on RNA interference: From nanoformulations to *in vivo* delivery. Nanotechnology.

[18] de Fougerolles A, Vornlocher HP, Maraganore J, Lieberman J (2007). Interfering with disease: A progress report on siRNA-based therapeutics. Nat. Rev. Drug Discov..

[19] Davis ME (2010). Evidence of RNAi in humans from systemically administered siRNA via targeted nanoparticles. Nature.

[20] Ron D, Walter P (2007). Signal integration in the endoplasmic reticulum unfolded protein response. Nat. Rev. Mol. Cell Biol..

[21] Mizushima N, Levine B, Cuervo AM, Klionsky DJ (2008). Autophagy fights disease through cellular self-digestion. Nature.

[22] Yan MM, Ni JD, Song D, Ding M, Huang J (2015). Interplay between unfolded protein response and autophagy promotes tumor drug resistance (Review). Oncol. Lett..

[23] Park J (2004). Ultra-large-scale syntheses of monodisperse nanocrystals. Nat. Mater..

[24] Zhang M, Cushing BL, O’Connor CJ (2008). Synthesis and characterization of monodisperse ultra-thin silica-coated magnetic nanoparticles. Nanotechnology.

