# Supplementary material for: Selective Capture and Purification of MicroRNAs and Intracellular Proteins through Antisense-vectorized Magnetic Nanobeads
Source: Sci Rep. 2019 Feb 14;9:2069. doi: 10.1038/s41598-019-39575-7 (PMC6375918; doi:10.1038/s41598-019-39575-7)
Supplement: Supplementary file 1 — Supplementary Information [file 41598_2019_39575_MOESM1_ESM.pdf]

Supporting information

Selective Capture and Purification of MicroRNAs and Intracellular Proteins through Antisense-vectorized Magnetic Nanobeads

Isabel Gessner[1], Xiaojie Yu[2,3], Christian Jüngst[4], Annika Klimpel[5], Lingyu Wang[2,3], Thomas Fischer[1], Ines Neundorf[5], Astrid Schauss[4], Margarete Odenthal[2,3,6], Sanjay Mathur[1,*]

[1] Institute of Inorganic Chemistry, University of Cologne, Greinstr. 6, 50939 Cologne, Germany

[2] Institute for Pathology, University Hospital of Cologne, Kerpener Str. 62, 50924 Cologne, Germany

[3] Center for Molecular Medicine (CMMC), University of Cologne, Robert-Koch-Straße 21, 50931 Cologne, Germany

[4] Cluster of Excellence - Cellular Stress Responses in Aging-Associated Diseases (CECAD), Imaging Facility, University of Cologne, Joseph-Stelzmann-Str. 26, 50931 Cologne, Germany

[5] Institute of Biochemistry, University of Cologne, Zuelpicher Str. 47, 50674 Cologne, Germany

[6] Center of Integrative Oncology, University Clinic of Cologne and Bonn, Germany

*corresponding author: sanjay.mathur@uni-koeln.de

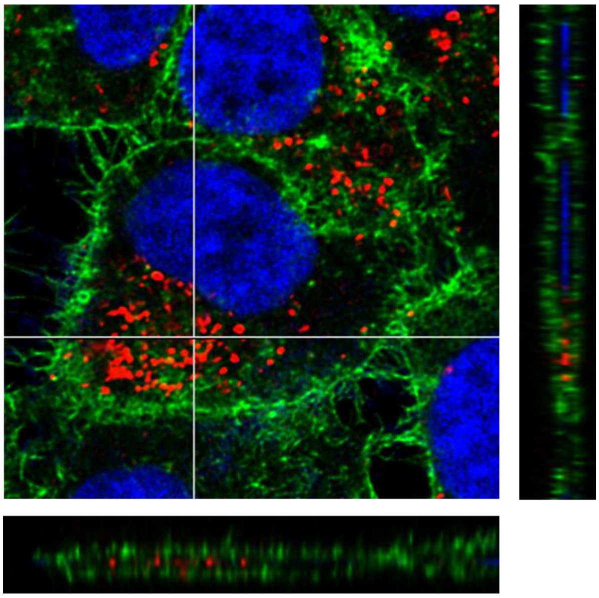

Figure S 1: Orthogonal view of confocal microscopic stackings performed for scramble functionalized magnetic beads in Huh7 cells.

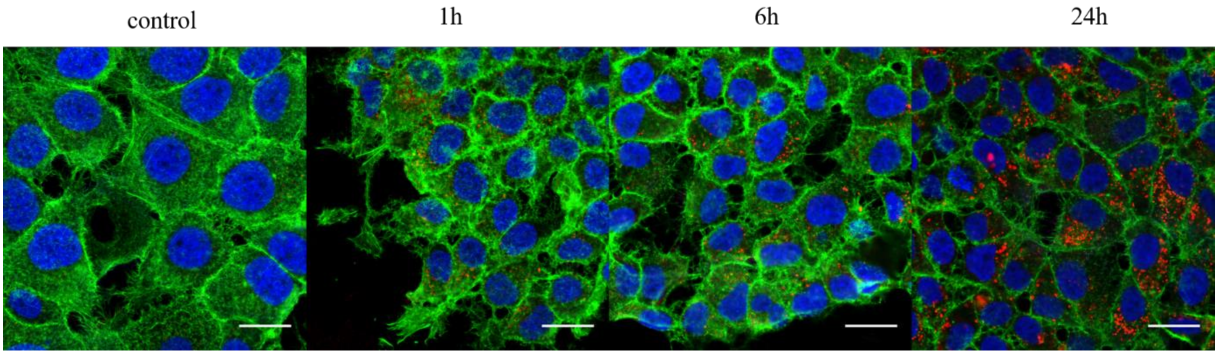

Figure S 2: Confocal microscopic images of Huh7 cells incubated with scramble functionalized magnetic beads for incubation times between 0-24 hours. The cytoskeleton was stained with phalloidin green, while the nucleus was stained with DAPI. The scale bar in all images refers to 25 µm.
